# Biofunctionalization strategies on tantalum-based materials for osseointegrative applications

**DOI:** 10.1007/s10856-015-5445-z

**Published:** 2015-02-11

**Authors:** Carlos Mas-Moruno, Beatriz Garrido, Daniel Rodriguez, Elisa Ruperez, F. Javier Gil

**Affiliations:** 1 Biomaterials, Biomechanics and Tissue Engineering Group, Department of Materials Science and Metallurgical Engineering, Technical University of Catalonia (UPC), ETSEIB, Av. Diagonal 647, 08028 Barcelona, Spain; 2 Biomedical Research Networking Centre in Bioengineering, Biomaterials and Nanomedicine (CIBER-BBN), Av. Diagonal 647, 08028 Barcelona, Spain; 3 Centre for Research in NanoEngineering (CRNE) - UPC, C/Pascual i Vila 15, 08028 Barcelona, Spain

## Abstract

The use of tantalum as biomaterial for orthopedic applications is gaining considerable attention in the clinical practice because it presents an excellent chemical stability, body fluid resistance, biocompatibility, and it is more osteoconductive than titanium or cobalt-chromium alloys. Nonetheless, metallic biomaterials are commonly bioinert and may not provide fast and long-lasting interactions with surrounding tissues. The use of short cell adhesive peptides derived from the extracellular matrix has shown to improve cell adhesion and accelerate the implant’s biointegration in vivo. However, this strategy has been rarely applied to tantalum materials. In this work, we have studied two immobilization strategies (physical adsorption and covalent binding via silanization) to functionalize tantalum surfaces with a cell adhesive RGD peptide. Surfaces were used untreated or activated with either HNO_3_ or UV/ozone treatments. The process of biofunctionalization was characterized by means of physicochemical and biological methods. Physisorption of the RGD peptide on control and HNO_3_-treated tantalum surfaces significantly enhanced the attachment and spreading of osteoblast-like cells; however, no effect on cell adhesion was observed in ozone-treated samples. This effect was attributed to the inefficient binding of the peptide on these highly hydrophilic surfaces, as evidenced by contact angle measurements and X-ray photoelectron spectroscopy. In contrast, activation of tantalum with UV/ozone proved to be the most efficient method to support silanization and subsequent peptide attachment, displaying the highest values of cell adhesion. This study demonstrates that both physical adsorption and silanization are feasible methods to immobilize peptides onto tantalum-based materials, providing them with superior bioactivity.

## Introduction

Metallic biomaterials are nowadays commonly used for bone replacing applications due to their unique combination of optimal mechanical properties, resistance to corrosion in biological environments and excellent biocompatibility [1, 2]. This alliance of properties has been described for stainless steel, cobalt–chromium (Co–Cr) alloys and titanium (Ti). In particular, Ti and its alloys (e.g. Ti–6Al–4V) are currently the major choice for dental and orthopedic applications [3]. Another biomaterial that is attracting a great deal of attention from both researchers and clinicians is tantalum (Ta). Ta unites mechanical strength, ductility and high chemical stability with an outstanding in vitro and in vivo biocompatibility, and very good osteoconductivity [4–7], thus offering interesting potential for orthopedic reconstructive applications. Moreover, in vivo studies have demonstrated no dissolution of Ta metal after several weeks of implantation and no evidence of inflammatory reaction was detected in tissues surrounding Ta implants [5].

Nevertheless, the use of Ta as implant material has been limited because of its elevated cost of production and difficult processing: it has a high melting point and it easily reacts with oxygen. Its high density is also a major drawback, preventing the elaboration of massive implants. For this reason, many studies have focused on the deposition of thin films of Ta onto other surfaces to confer its excellent properties to these materials without increasing their density. In this regard, the deposition of Ta coatings onto metallic substrates has been shown to improve the corrosion resistance and biocompatibility of stainless steel [8], Co–Cr alloys [9] and Ti-based materials [10]. Interestingly, Ta coatings on Ti/TiO_2_ surfaces were shown to improve the adhesion and proliferation of human osteoblasts [11], as well as their production of alkaline phosphatase and mineralization [12], compared to untreated Ti. Likewise, in a series of recent studies the osteogenic differentiation of human mesenchymal stem cells was significantly enhanced on Ta surfaces in comparison with Ti surfaces [13–15].

Furthermore, the introduction of porous Ta implants (80–85 % porosity), which show an elastic modulus of ~3 GPa (i.e. very close to that of trabecular bone) [16], represents a powerful alternative to classical metallic implants because it facilitates implant stability and allows a closer contact between the implant and living tissues [17–19]. The favorable pore size and the desirable biomechanical compatibility of porous Ta has resulted in numerous applications in joint replacements such as knee [20–22], hip [23–25] and shoulder [26].

Besides the excellent mechanical and biological properties exhibited by Ti and Ta, the success of these materials as orthopedic and/or dental implants relies on their capacity to establish an optimal osseointegration with peri-implant bone right after the implant surgery [27]. However, both Ti and Ta are biologically inert materials and in vivo may not elicit the specific cellular responses required for a fast and reliable bone regeneration. Such minimal biological interaction with the surrounding tissues might jeopardize the long-term stability of the implant, especially in patients with compromised clinical scenarios [1].

Thus, surface modifications aiming at increasing the bioactivity of implant materials are regarded as promising approaches to accelerate their osseointegrative capacity [1, 28–30]. In regard to this, the immobilization of cell adhesive molecules from the extracellular matrix (ECM) onto Ti-based materials has been thoroughly investigated for repairing and regenerating bone tissues, with encouraging outcomes both in vitro and in vivo [1, 30–32]. Such biomimetic strategies to functionalize Ti include the use of native ECM proteins and their recombinant fragments [33–36], peptides [37–40] and peptidomimetics [41–43].

However, this strategy has been rarely applied to Ta materials. Whereas NaOH/thermal treatments (i.e. bone-like apatite formation) [44, 45] and coatings/growth of calcium phosphate layers [46–48] onto Ta substrates have been vastly explored, the literature has only documented a few examples on the biofunctionalization of Ta with cell adhesive ECM molecules. The physical adsorption of fibronectin on nanostructured Ta surfaces efficiently enhanced the proliferation of mesenchymal stem cells compared to uncoated samples [49]. A positive effect on the proliferation of this cell type was also observed onto tantalized steel surfaces upon covalent immobilization of type-I collagen [8]. Nonetheless, the only report studying the functionalization of Ta surfaces with a synthetic cell adhesive peptide corresponds to a recent work from McNichols et al., in which Ta substrates were coated with a cyclic RGD peptide to improve vascular endothelialization [50]. To the best of our knowledge, the use of RGD peptides for osseointegrative applications on Ta has not yet been investigated.

In this work, we proposed the biofunctionalization of Ta surfaces with a synthetic cell adhesive peptide (i.e. an RGD peptide) as a feasible and inexpensive approach to increase the bioactivity of this material and thus improve its efficacy for application in bone regeneration. In addition, the use of short synthetic peptides offers several advantages over the use of proteins, including higher stability to temperature and pH changes, lack of immunogenicity, ease of preparation, well-defined chemical compositions, and capacity to be immobilized on surfaces at high densities with an optimal orientation [1, 51, 52].

Thus, we have investigated the activation and biofunctionalization of Ta surfaces with a short synthetic RGD peptide by either physical adsorption or covalent binding via silane chemistry. These two methods of immobilization have been physicochemically characterized at the surface level by means of contact angle measurements, surface energy calculations, white light interferometry, scanning electron microscopy (SEM) and X-ray photoelectron spectroscopy (XPS). The biological performance of the resulting surfaces has also been evaluated by cell adhesion studies using human osteogenic sarcoma (Saos-2) cells.

## Materials and methods

### Biofunctionalization of Ta samples

#### Preparation of Ta disks

Ta disks (3 mm thick, 6.4 mm diameter) were obtained from Ta bars of 99.95 % purity (Alfa Aesar, Karlsruhe, Germany). Samples were polished to achieve mirror-like, smooth surfaces, (average surface roughness, R_a_, ≈40 nm) by grinding with abrasive SiC papers (Presi, Oxford, UK) of decreasing grit size (from P800 to P4000—European P-grade standard), followed by polishing with a diamond suspension (Presi) (1.0 µm particle size) on cotton clothes. After polishing, samples were ultrasonically rinsed with cyclohexane, isopropanol, distilled water, ethanol and acetone, and stored dry under vacuum.

#### Synthesis of the cell-adhesive peptide

**Fig. 1:**
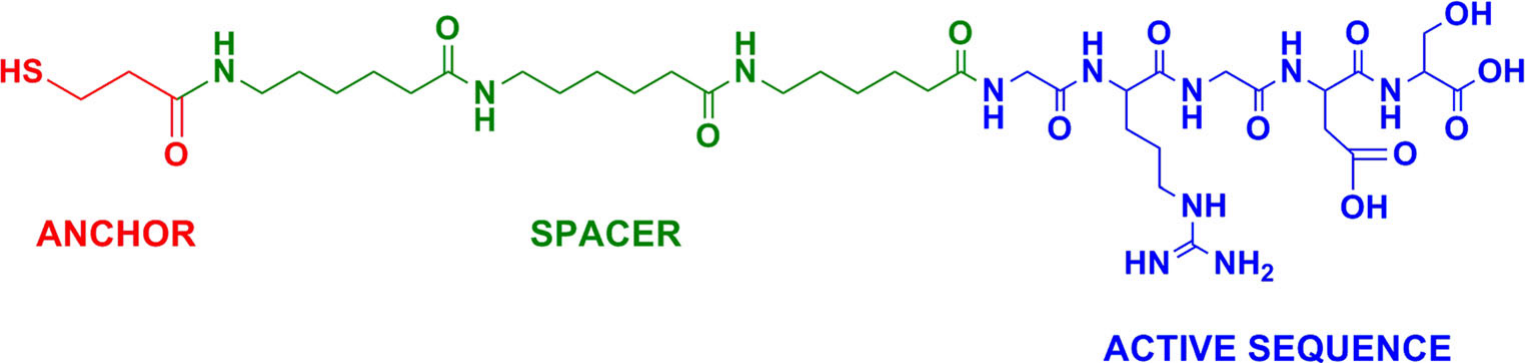
Chemical structure of the cell adhesive RGD peptide

The cell adhesive peptide RGD, which comprises the active sequence Gly-Arg-Gly-Asp-Ser (GRGDS) as active domain, three units of 6-aminohexanoic acid (Ahx) as spacer [38] and 3-mercaptopropionic acid (MPA) as anchoring moiety (MPA-Ahx-Ahx-Ahx-GRGDS-OH, Fig. 1), was manually synthesized in solid-phase following the Fmoc/tBu strategy and using 2-chlorotrityl chloride resin (200 mg, loading of 1.0 mmol/g) (Iris Biotech GmbH, Marktredwitz, Germany) as previously reported [40]. The purified peptide was characterized as follows: HPLC (XBridge BEH130 C-18 column, 10–40 % ACN over 8 min, tR = 4.255 min, purity 97 %), MALDI-TOF (m/z calcd. for C_38_H_67_N_11_O_13_S: 917.46, found: 918.30 [M + H]^+^, 940.28 [M + Na]^+^, 959.25 [M + K]^+^).

#### Activation of samples

Prior to functionalization, samples were either passivated with HNO_3_ or activated by UV/ozone treatment. *Passivation with HNO*_*3*_: Ta samples were immersed in a 32.5 % (v/v) solution of HNO_3_ and treated for 10 min under sonication. After this treatment, samples were thoroughly washed with distilled water, ethanol and acetone, and dried with nitrogen gas. *UV/ozone treatment*: samples were placed in a UVO-Cleaner^®^ (model 42-220, Jelight Company, Inc., Irvine, CA, USA) and treated with UV/ozone for 2 h. Samples were then kept under vacuum.

#### Silanization of the samples

Activated samples were silanized with (3-aminopropyl)triethoxysilane (APTES) (2 %, v/v) (Sigma-Aldrich, St Louis, MO, USA) in anhydrous toluene for 1 h at 70 °C under nitrogen atmosphere. After this time, Ta disks were subjected to sonication for 10 min to remove non-covalently bound silanes, and washed with toluene, isopropanol, distilled water, ethanol and acetone, and dried with nitrogen. Aminosilanized samples were then further modified by reaction with 2 mg/mL of the bifunctional crosslinker 3-maleimidopropionic acid *N*-hydroxysuccinimide ester (Alfa Aesar) in *N*,*N*-dimethylformamide (DMF) for 1 h at room temperature. Samples were finally washed with DMF, distilled water, ethanol and acetone, and dried with nitrogen. This method was adapted, with some modifications, from previously published protocols [53, 54].

#### Immobilization of RGD peptide onto Ta samples

**Fig. 2:**
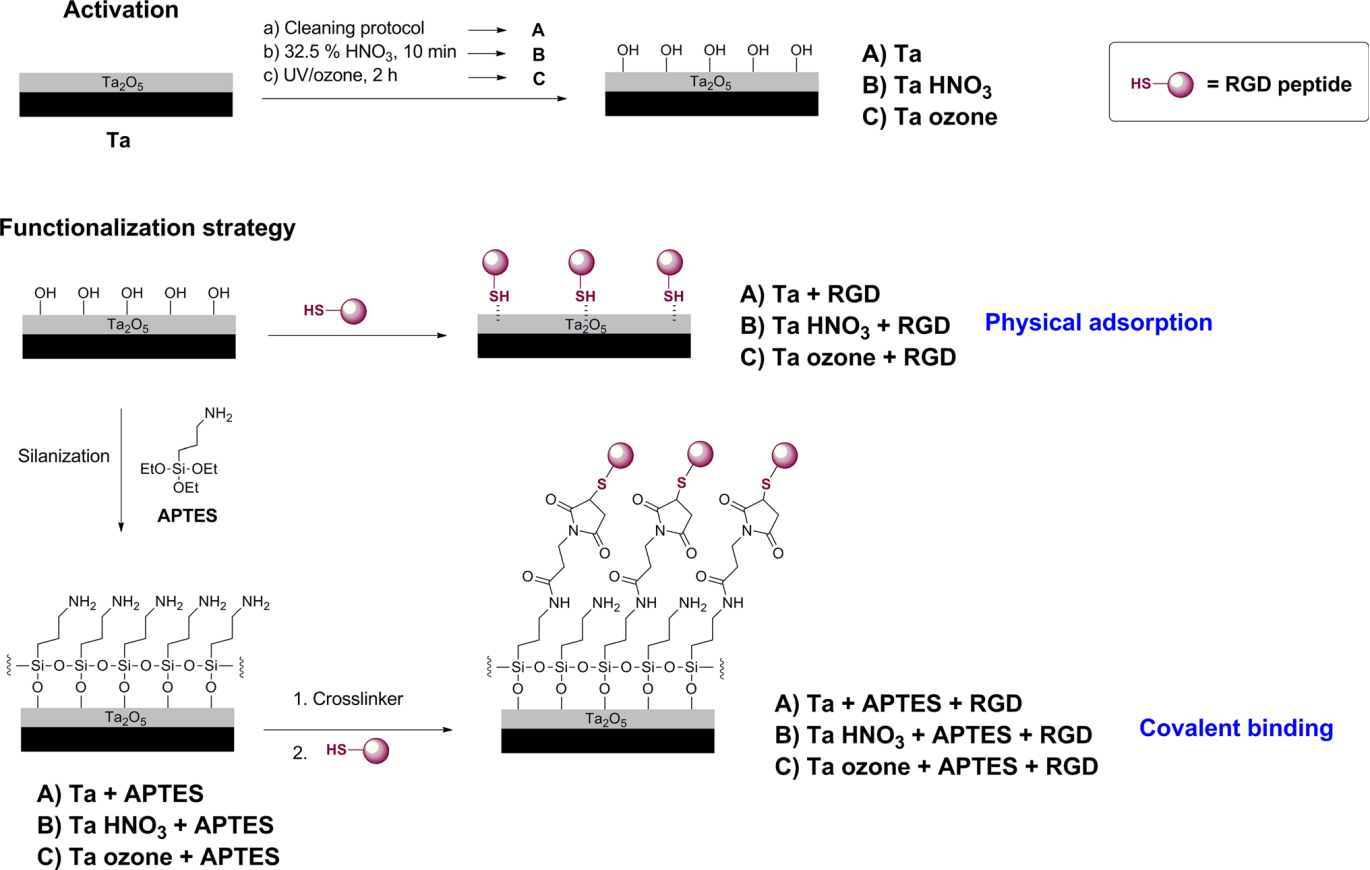
Summary of the immobilization strategies used to biofunctionalize Ta samples

For aminosilanized samples with APTES, the RGD peptide was dissolved in phosphate buffered saline (PBS) at pH 6.5 at a 100 µM concentration, and deposited onto the Ta samples (100 µL/disk) overnight at room temperature. To physically adsorb the peptide on non-silanized samples, the same conditions were used but using PBS at pH 7.4 instead. Control samples were only treated with buffer. After peptide incubation, samples were gently washed with PBS and dried with nitrogen. The biofunctionalized samples, and their controls, are codified as follows (Fig. 2):

- Ta: Non-treated tantalum
- Ta HNO_3_: Tantalum passivated with nitric acid
- Ta ozone: Tantalum treated with UV/ozone
- Ta + RGD: Tantalum coated with 100 µM of RGD peptide
- Ta HNO_3_ + RGD: Tantalum passivated with nitric acid and coated with 100 µM of RGD peptide
- Ta ozone + RGD: Tantalum treated with UV/ozone and coated with 100 µM of RGD peptide
- Ta + APTES: Tantalum silanized with APTES
- Ta HNO_3_ + APTES: Tantalum passivated with nitric acid and silanized with APTES
- Ta + ozone + APTES: Tantalum treated with UV/ozone and silanized with APTES
- Ta + APTES + RGD: Tantalum silanized with APTES and coated with 100 µM of RGD peptide
- Ta HNO_3_ + APTES + RGD: Tantalum passivated with nitric acid, silanized with APTES and coated with 100 µM of RGD peptide
- Ta + ozone + APTES + RGD: Tantalum treated with UV/ozone, silanized with APTES and coated with 100 µM of RGD peptide

### Physicochemical characterization of biofunctionalized samples

#### Static contact angle measurements and surface energy calculations

Static contact angle measurements on Ta surfaces were performed using a Contact Angle System OCA15 plus (Dataphysics, Filderstadt, Germany) with the sessile drop method. All measurements were done at room temperature using ultrapure Milli-Q water and diiodomethane as wetting liquids (drop volume of 0.5 µL). Static contact angles were calculated using a Laplace–Young fitting with SCA 20 software (Dataphysics). Contact angle values presented here represent the mean of three measurements per disk for three sample replicates. The surface energy and its dispersive and polar components were determined using the Young–Laplace (1) and Owen–Wendt (2) equations.

1 $$\gamma_{S} = \gamma_{SL} + \gamma_{L} \cos \theta$$

2 $$\gamma_{L} \left( {1 + \cos \theta } \right) = 2\left( {\left( {\gamma_{L}^{d} \gamma_{S}^{d} } \right)^{{{\raise0.7ex\hbox{$1$} \!\mathord{\left/ {\vphantom {1 2}}\right.\kern-0pt} \!\lower0.7ex\hbox{$2$}}}} + \left( {\gamma_{L}^{p} \gamma_{S}^{p} } \right)^{{{\raise0.7ex\hbox{$1$} \!\mathord{\left/ {\vphantom {1 2}}\right.\kern-0pt} \!\lower0.7ex\hbox{$2$}}}} } \right)$$

where γ_S_ is the surface tension of the solid (S), γ_L_ the surface tension of the liquid (L), γ_SL_ the interfacial free energy or surface energy between L and S, θ the contact angle between L and S, and γ^d^ and γ^p^ represent the dispersive and polar components of the surface energy, respectively.

#### Topographical analysis

The topographical features (morphology and roughness) of the samples were studied by means of scanning electron microscopy (SEM) and white light interferometry. SEM analysis was conducted on a Zeiss Neon40 microscope (Carl Zeiss, Jena, Germany). For each sample, five images were taken at a working distance of 7 mm and a potential of 5 kV. The surface roughness of the samples was determined by interferometry using a Wyko NT9300 Optical Profiler microscope (Veeco Instruments, New York, NY, USA) in vertical scanning interferometry mode. Data analysis was performed with Wyko Vision 4.10 software (Veeco Instruments). The average roughness (R_a_) was measured by triplicate for each sample. For each surface treatment three disks were analyzed.

#### X-ray photoelectron spectroscopy (XPS)

The chemical composition of the functionalized Ta samples was analyzed using an XPS equipment (SPECS Surface Nano Analysis GmbH, Berlin, Germany) with a Mg anode XR50 source operating at 150 W and a Phoibos 150 MCD-9 detector. High resolution spectra were recorded with a pass energy of 25 eV at 0.1 eV steps at a pressure below 7.5 × 10^−9^ mbar. Binding energies were referred to the C 1 s signal at 284.8 eV. Each sample series was studied by duplicate. Data was analyzed using CasaXPS software (Version 2.3.16, Casa Software Ltd., Teignmouth, UK).

### Biological characterization of biofunctionalized samples

#### Cell culture

Cellular experiments were conducted using the human osteogenic sarcoma (Saos-2) cell line as osteoblast-like cellular model. Saos-2 cells were cultured in McCoy’s 5A medium (Sigma-Aldrich) supplemented with 10 % (v/v) fetal bovine serum (FBS), 1 M 4-(2-hydroxyethyl)-1-piperazineethanesulfonic acid (HEPES), 1 % (w/v) sodium pyruvate, 50 U/mL penicillin, 50 μg/mL streptomycin and 1 % (w/v) l-glutamine. Cells were maintained at 37 °C, in a humidified atmosphere containing 5 % (v/v) CO_2_, changing culture medium every 2–3 days. Upon reaching confluence, cells were detached by trypsin–EDTA and subcultured into a new flask. All experiments were performed using cells at passages between 25 and 35.

#### Cell adhesion studies: number of cells attached

Functionalized Ta samples were transferred into 48-well plates and blocked with 1 % (w/v) bovine serum albumin (BSA) in PBS for 40 min at room temperature. This step was done to reduce non-specific interactions between the cells and the surface. Next, Saos-2 cells were seeded at a density of 50,000 cells/mL (500 µL/disk) and allowed to attach in serum free medium. After 4 h of incubation at 37 °C, samples were rinsed twice with PBS to remove non-adherent cells. To determine the number of adherent cells, cells were lysed with 300 µL/disk of mammalian protein extraction reagent (M-PER) and the activity of lactate dehydrogenase (LDH) enzyme was measured by means of a conventional colorimetric assay (Cytotoxicity Detection Kit (LDH), Roche Diagnostics, Mannheim, Germany) using a multimode microplate reader (Infinite M200 PRO, Tecan Group Ltd., Männedorf, Switzerland). To convert the absorbance read-out of the test into cell numbers, a standard curve of defined cell concentrations was applied.

#### Cell adhesion studies: immuno-staining of nuclei and actin fibers

Saos-2 cells were incubated for 4 h onto Ta samples as explained above. After this time, cells attached to the surfaces were fixed for 30 min with 4 % (w/v) paraformaldehyde (PFA). Next, cells were permeabilized with 500 µL/disk of 0.05 % (w/v) triton X-100 in PBS for 20 min, and blocked with 1 % BSA (w/v) in PBS for 30 min. Washings between steps were all performed with PBS-Gly (PBS containing 20 mM of glycine) for 3 × 5 min. Then, 100 µL/disk of phalloidin-rodhamine (1:300) were incubated in triton 0.05 % (w/v) in PBS for 1 h in the dark. In a final step, nuclei of cells were also stained with 500 µL/disk of 4′,6-diamidino-2-phenylindole (DAPI, 1:1,000) in PBS-Gly for 2 min in the dark. Metallic disks were then mounted on microscope slides and analyzed by fluorescence microscopy (Nikon E600, Tokyo, Japan). The spreading of cells attached on each surfaces was assessed using ImageJ 1.46R software (NIH, Bethesda, MD, USA). Spreading of adherent cells was measured for at least 10 cells for each sample and averaged for three samples for each condition. All cellular studies were done using triplicates and repeated at least in two independent assays to ensure reproducibility.

### Statistical analysis

All data presented in this study are given as mean values ± standard deviations. Significant differences between group means were analyzed either by ANOVA test followed by post hoc pairwise comparisons using Tukey’s test, or by Kruskal–Wallis non-parametric test followed by Mann–Whitney test. Confidence levels were set at 95 % unless otherwise stated.

## Results and discussion

### Functionalization strategy

The aim of the present work was to investigate and characterize the biofunctionalization of Ta surfaces with a well-known cell adhesive peptide in order to enhance the adhesion of osteoblast-like cells onto these materials. Improving the bioactivity of Ta holds great potential to improve the biological performance of Ta-based implant materials. As previously introduced, such approach, widely studied on Ti and other materials, has rarely been explored on Ta substrates.

To establish a reliable functionalization protocol to immobilize bioactive peptides, two classical approaches were considered: physical adsorption and covalent binding via silanization (Fig. 2). Physical adsorption is a simple and inexpensive method, and usually does not require to chemically modify the surface of the material. Its main disadvantage, though, is that the binding of synthetic oligopeptides by physisorption is commonly less stable than that achieved by covalent methods such as silanization, in which the peptides are irreversibly bound to the surfaces [32, 39]. Silanization, however, often requires the activation of the surface to generate accessible hydroxyl groups that will allow a successful binding and polymerization of siloxane layers. To this end, we subjected Ta samples to either passivation with HNO_3_ [8] or UV/ozone treatment [48]. Both methods do not modify the topography (i.e. roughness) of the surfaces, an advantage over other activation methods that use stronger acids or alkaline etchings [29]. Silanization was performed with APTES following well-established protocols [53, 54]. The conditions used in this study were optimized to yield a sub- to monolayer of silane on the surface [55, 56]. The reactive amino groups of the silane layer were further modified with 3-maleimidopropionic acid *N*-hydroxysuccinimide ester. The presence of maleimide groups on the surfaces is useful to attach thiol-bearing peptides through a Michael addition (Fig. 2). As a model of cell-adhesive peptide, the well-known RGD sequence was selected [57]. This ECM-motif has affinity for integrin receptors expressed in a large number of cells and has been described to efficiently promote cell adhesion and proliferation [31, 52, 58]. In our study, the RGD peptide was designed with three main characteristics: The bioactive sequence, an Ahx-based spacer, and an anchoring unit (Fig. 1). The spacer system is important to ensure an adequate accessibility of the RGD motif and efficiently interact with cell receptors (i.e. integrins) [38, 43]. The MPA group has a thiol functionality that can chemoselectively bind to maleimide groups, thereby providing a selective and stable anchorage to the surfaces.

### Physicochemical characterization of the surfaces

**Fig. 3:**
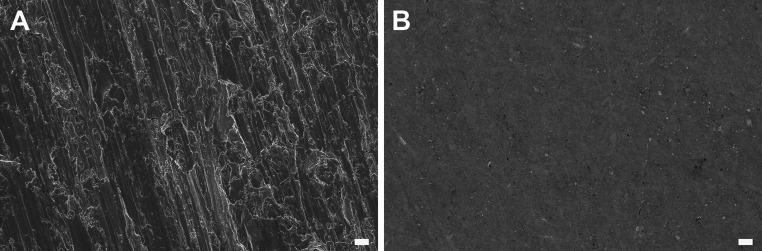
Scanning electron microscope images. **a** Trimmed Ta samples before polishing. **b** Smooth Ta samples after polishing. Surface activation treatments rendered similar micrographs as observed for smooth control samples (**b**). *Scale bar* = 2 µm

The effect of the polishing protocol and the activation methods on the topography of Ta surfaces was analyzed by white light interferometry and SEM. As observed in Table 1, after grinding and polishing of trimmed samples, the average roughness of Ta disks was significantly reduced to values of ~40 nm, corresponding to smooth, mirror-like surfaces. As anticipated, the activation methods did not significantly modify this parameter. Consistent with these values, visualization of trimmed samples by SEM revealed a very irregular surface, with marked grooves and crevices and many topographical peaks and valleys (Fig. 3a). After polishing, a smooth and homogeneous surface was obtained with no relevant topographical features (Fig. 3b). This morphology was not altered by the activation methods. Thus, no effects on cell adhesion are expected based on this factor [59, 60].

**Table 1:** Average roughness values (Ra) of Ta surfaces

Surface^a^	Trimmed	Ta	Ta HNO_3_	Ta ozone
Ra (nm)	912 ± 172 (*)	40.6 ± 8.0	47.9 ± 6.3	45.5 ± 8.2

Values are expressed as mean ± standard deviation

^a^Surfaces are designated as follows: Ta disks before grinding and polishing (Trimmed); Ta disks polished (Ta); Ta disks polished and passivated with HNO_3_ (Ta HNO_3_); Ta disks polished and UV/ozone-treated (Ta ozone)

* Statistically significant differences (P < 0.05) were observed for Trimmed versus other samples

**Fig. 4:**
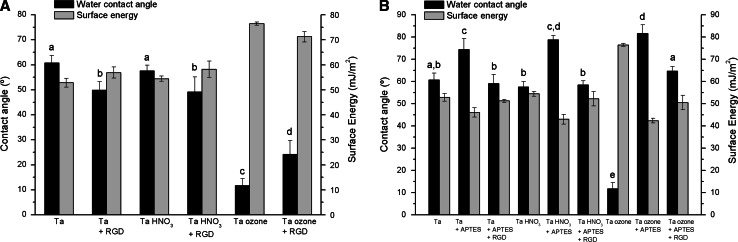
Water contact angle measurements and surface energy calculations. **a** Activation of Ta samples (Ta) by passivation (Ta HNO_3_) or UV/ozone treatment (Ta ozone), and physical adsorption of RGD peptide (+RGD). **b** Activation of Ta samples (Ta) by passivation (Ta HNO_3_) or UV/ozone treatment (Ta ozone), silanization (+APTES) and covalent attachment of RGD peptide (+RGD). Values are expressed as mean ± standard deviation. *Letters* (*a–e*) denote statistically significant differences (P < 0.05) between groups

The hydrophilicity of the samples (i.e. wettability) and surface energy was investigated by contact angle measurements. These physicochemical properties are key parameters affecting the adsorption of biomolecules and short-term cell adhesive events [29, 61]. Furthermore, changes in contact angle values can be used to monitor each step of the functionalization process (Fig. 4).

As shown in Fig. 4a, oxidation of Ta surfaces with HNO_3_ did not significantly modify the water contact angle of Ta samples. In contrast, activation with ozone drastically reduced this value (P < 0.05). This observation indicates that UV/ozone treatments generate highly hydrophilic surfaces, most likely by efficiently removing hydrophobic contaminants from the surface and by increasing the number of accessible hydroxyl groups. Such argumentation is in agreement with the increase detected in surface energy (mainly in its polar component, not shown). Physical adsorption of the RGD peptide on Ta and HNO_3_-treated Ta surfaces increased their wettability to a similar extent (contact angles around 50°). On the contrary, the peptide decreased the wettability of the highly hydrophilic ozone-treated Ta surfaces. Still, the lowest contact angle values were detected for this surface (contact angle below 25°, Ta ozone + RGD versus other samples, P < 0.05). From this analysis we can conclude:

(i) Upon peptide binding, Ta/Ta HNO_3_ surfaces render surfaces with very similar hydrophilicity. This observation could be related to a similar extent of peptide attachment on these surfaces
(ii) On the contrary, the binding of the RGD peptide on Ta ozone surfaces yields surfaces that are much more hydrophilic. This effect could be attributed to lower peptide coverage, due to electrostatic repulsions between these highly negatively charged Ta surfaces and the peptides, which bear an overall negative charge (−1) under the coating conditions (pH 7.4) [28, 39].

The covalent binding of the RGD peptide through silanization was also studied with contact angle measurements (Fig. 4b). The introduction of APTES resulted in a clear increase in the contact angle of all samples (P < 0.05), in agreement with the hydrophobic nature of silane molecules. This effect was more remarkable for ozone-treated samples. These results suggest that UV/ozone treatments might be more effective in generating hydroxyl groups on the surface than the other methods, therefore yielding a most efficient silanization. In addition, the positive charge present in APTES molecules may promote in this case a higher number of electrostatic interactions with surface silanol groups [56]. The attachment of the RGD peptide on silanized samples decreased their contact angle values to a similar level for all conditions (though slightly higher values were observed on ozone-treated samples, P < 0.05). Thus, the amount of peptide bound on the surfaces via silanization might be similar for Ta + APTES + RGD and Ta HNO_3_ + APTES + RGD, but slightly higher for Ta ozone + APTES + RGD.

**Fig. 5:**
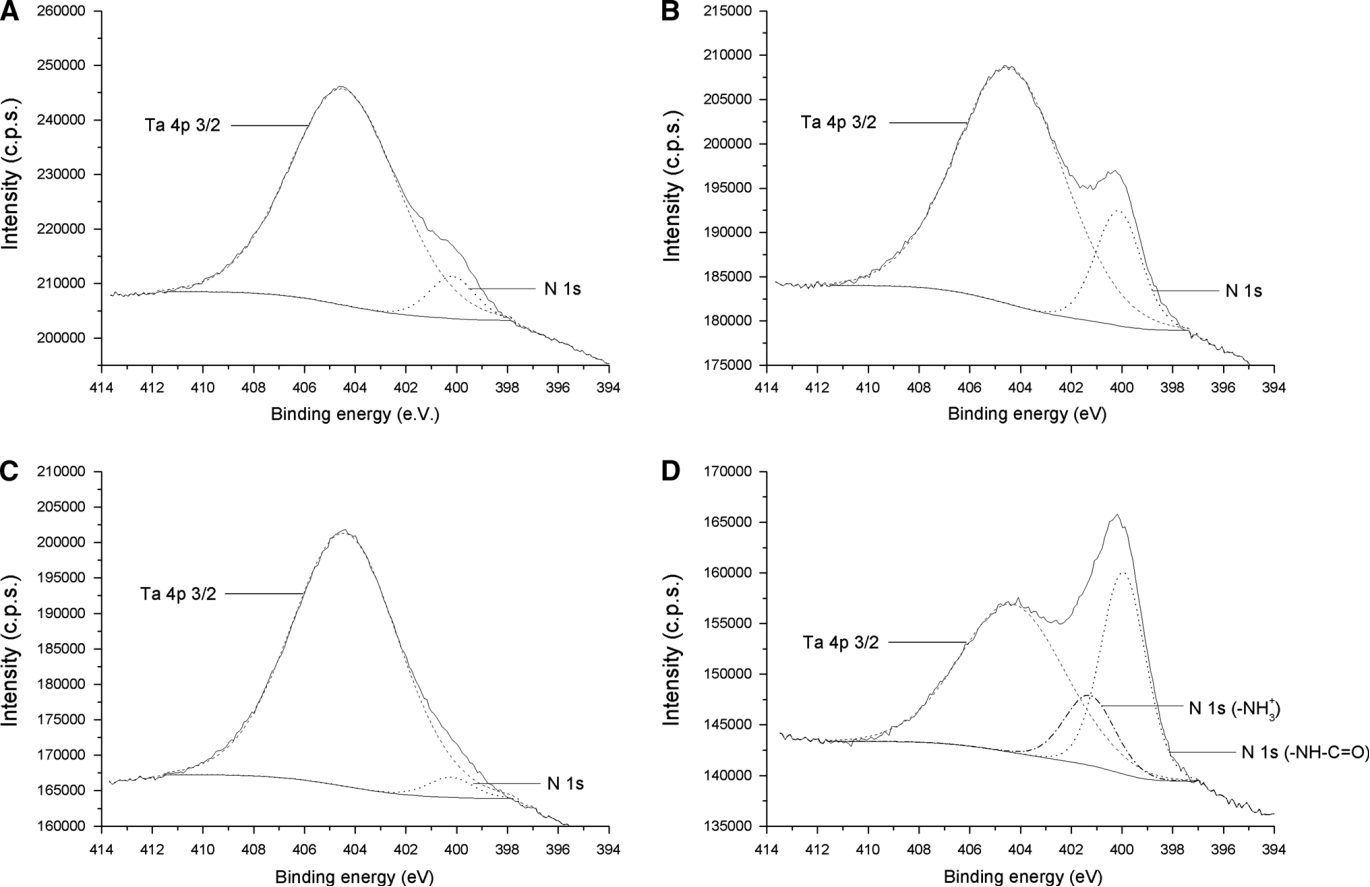
Curve-fitting deconvolution of high resolution XPS spectra (Ta 4p 3/2 and N 1s signals). **a** Control Ta samples (Ta). **b** Physical adsorption of RGD peptide on control surfaces (Ta + RGD). **c** Physical adsorption of RGD peptide on UV/ozone-treated surfaces (Ta ozone + RGD). **d** Covalent binding of RGD peptide via silanization on UV/ozone-treated surfaces (Ta ozone + APTES + RGD)

To further corroborate these findings, the chemical composition of the surfaces was characterized by XPS (Table 2). Control Ta surfaces displayed the expected Ta 4f and O 1s signals corresponding to Ta_2_O_5_. The high percentage of C 1s is commonly attributed to atmospheric contaminants. A minor amount of N 1s and Si 2p were also detected. The presence of Si corresponds to SiC incrustations from the grinding process, as determined by energy-dispersive X-ray spectroscopy during SEM analysis. HNO_3_ treatment did not significantly alter the chemical composition of Ta. On the contrary, UV/ozone activation clearly reduced the C content and increased the detectable O 1s signal. Thus, it seems UV/ozone treatment efficiently removes hydrophobic contaminants from the surfaces, in agreement with the drastic reduction in water contact angle previously observed for these samples (Fig. 4a). Physical adsorption of RGD onto these surfaces followed two trends. In Ta and Ta HNO_3_ samples, the percentage of N was increased, together with a decrease in that of Ta. Both effects are typical indicators of peptide attachment [40, 53, 54]. These two surfaces showed almost identical chemical compositions, confirming contact angle data. On the contrary, the content of N remained unmodified when the peptide was incubated on Ta ozone samples. This result indicates a low efficiency in peptide attachment, as we anticipated based on wettability studies (Fig. 4a) and electrostatic repulsions. To illustrate this response, high resolution spectra of N 1s are shown in Fig. 5. Although the N 1s signal partially overlaps with the Ta 4p 3/2 curve, it is evident from the spectra analysis that the N 1 s signal increases upon binding of the RGD peptide on Ta surfaces (Fig. 5a vs. b), while no increase is observed onto ozone-treated samples (Fig. 5a vs. c). The process of silanization was analyzed according to the Si 2p signal. APTES binding seemed to be more efficient on ozone-treated samples (6.2 % Si) than on the other surfaces (3.6–4.1 % Si), which might be due to the higher hydrophilicity (i.e. hydroxyl groups) achieved by UV/ozone activation. Subsequent peptide attachment yielded significant increases in the percentages of N and C, and reduction in the detectable Ta_2_O_5_ signal, compared to control samples. The most remarkable effects were observed on ozone-treated surfaces, consisting with a more efficient silanization. Deconvolution of this signal resulted in two clear signals (Fig. 5d), one corresponding to free protonated amino groups of APTES (–NH_3_^+^, ~401 eV) and another corresponding to amide bonds of the peptide backbone (–NH–C=O, ~400 eV) [40, 53–55].

**Table 2:** Analysis of the chemical composition (atomic %) of Ta surfaces by XPS

Sample	Composition (atomic %)				
	C 1s	O 1s	N 1s	Si 2p	Ta 4f
Ta	32.8 ± 1.0	47.1 ± 2.2	1.6 ± 0.1	2.2 ± 0.2	16.3 ± 1.1
Ta HNO_3_	36.1 ± 8.3	45.5 ± 6.2	2.1 ± 0.1	2.4 ± 0.5	14.0 ± 2.4
Ta ozone	19.0 ± 1.1	60.3 ± 1.1	0.9 ± 0.2	1.9 ± 0.6	17.9 ± 0.5
Ta + RGD	36.2 ± 4.5	46.3 ± 3.7	3.1 ± 0.1	1.4 ± 0.5	13.1 ± 0.4
Ta HNO_3_ + RGD	36.3 ± 3.3	45.9 ± 3.8	3.0 ± 0.4	2.2 ± 0.0	12.7 ± 0.1
Ta ozone + RGD	25.5 ± 5.3	55.8 ± 3.9	0.9 ± 0.2	1.9 ± 0.3	15.8 ± 1.9
Ta + APTES + RGD	41.4 ± 0.3	39.8 ± 0.1	4.5 ± 0.2	4.1 ± 0.2	10.2 ± 0.1
Ta HNO_3_ + APTES + RGD	42.4 ± 3.0	39.1 ± 1.6	4.5 ± 0.4	3.6 ± 0.1	10.4 ± 0.9
Ta ozone + APTES + RGD	44.7 ± 1.2	35.5 ± 0.4	5.8 ± 0.2	6.2 ± 0.1	7.8 ± 0.4

Atomic percentages are expressed as mean ± standard deviation

Overall, XPS studies were proven useful to corroborate the data obtained by contact angle measurements. The most remarkable findings are:

(i) The RGD peptide can be physisorbed on Ta and HNO_3_-treated Ta surfaces, but this method of immobilization is not effective on very hydrophilic, ozone-activated samples.
(ii) The covalent attachment of the peptide through silanization is achieved on all surfaces, although this process seems to be more efficient on ozone-treated surfaces.

### Biological characterization of the surfaces: adhesion of osteoblast-like cells

The effect of the biofunctionalization strategies on the biological performance of Ta surfaces was analyzed by studying the adhesion of sarcoma osteogenic (Saos-2) cells to these substrates. The RGD sequence, originally identified in fibronectin [57], is a common cell adhesive sequence found in many proteins of bone ECM. This cell-binding domain interacts with integrin receptors expressed by osteoblasts such as αvβ3, αvβ5 and α5β1 that trigger the adhesion, spreading and proliferation of these cells [31, 52, 58].

**Fig. 6:**
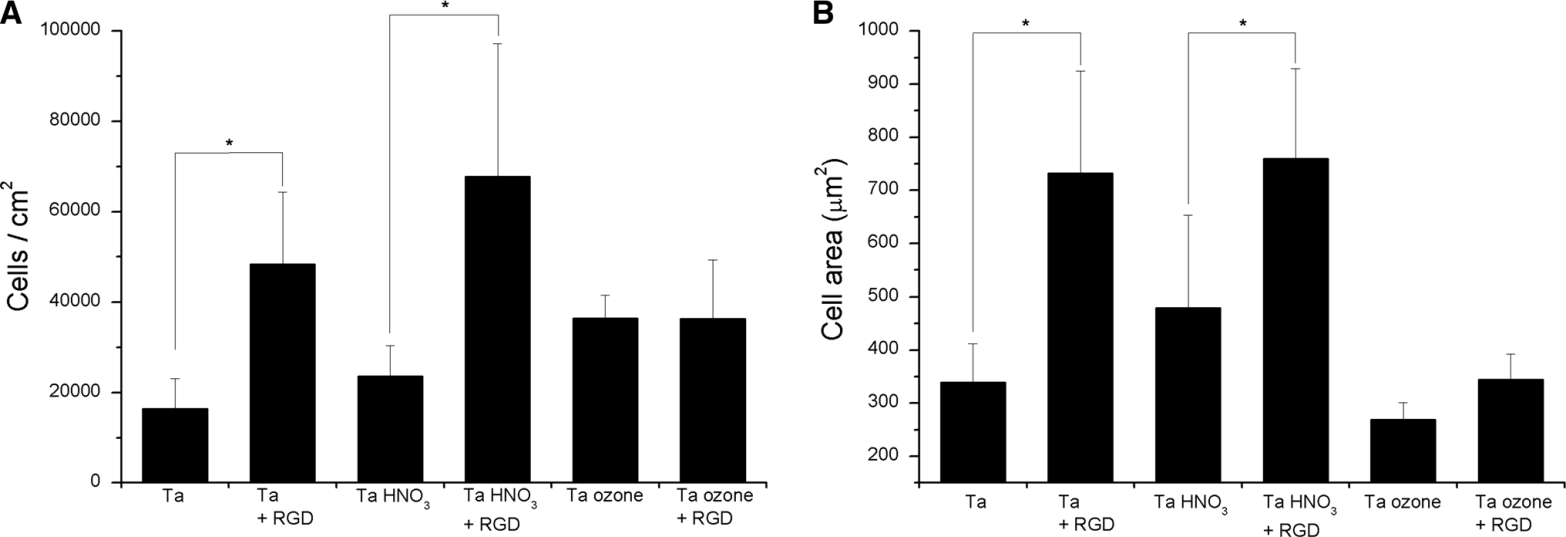
Adhesion of Saos-2 cells on Ta surfaces functionalized with the RGD peptide by physical adsorption. Cell adhesion was analyzed after 4 h of incubation. **a** Cell attachment (cells/cm^2^); and **b** cell spreading (cell area in µm^2^). Values are expressed as mean ± standard deviation. The symbol (*) denotes statistically significant differences between groups (cell numbers P < 0.1, cell area P < 0.05)

The physical adsorption of the RGD motif on control and HNO_3_-passivated Ta surfaces significantly increased (P < 0.1) the number of adherent cells after 4 h of incubation (Fig. 6a). Remarkably, this enhancement in cell adhesion was accompanied by a clear increase (P < 0.05) in the spreading (i.e. cell area) of adherent cells (Fig. 6b). Although Ta HNO_3_ + RGD samples showed the highest values of cell attachment, cell numbers were not statistically different than those of Ta + RGD. Thus the presence of the RGD sequence, as previously characterized by contact angle and XPS measurements, results in an effective improvement in the adhesion of Saos-2 cells on Ta surfaces. However, the effect of RGD physisorption on Ta samples activated with UV/ozone was totally different: any statistically significant difference was observed in terms of cell attachment and spreading between samples functionalized or not with the RGD peptide (Ta ozone vs. Ta ozone + RGD, Fig. 6). These results confirm our previous physicochemical characterization studies, which described a poor attachment of the RGD sequence in these sorts of activated-surfaces. Hence, UV/ozone treatment should be discarded as activating method for the physical adsorption of RGD peptides on Ta surfaces, especially if the peptides display an overall negative charge.

**Fig. 7:**
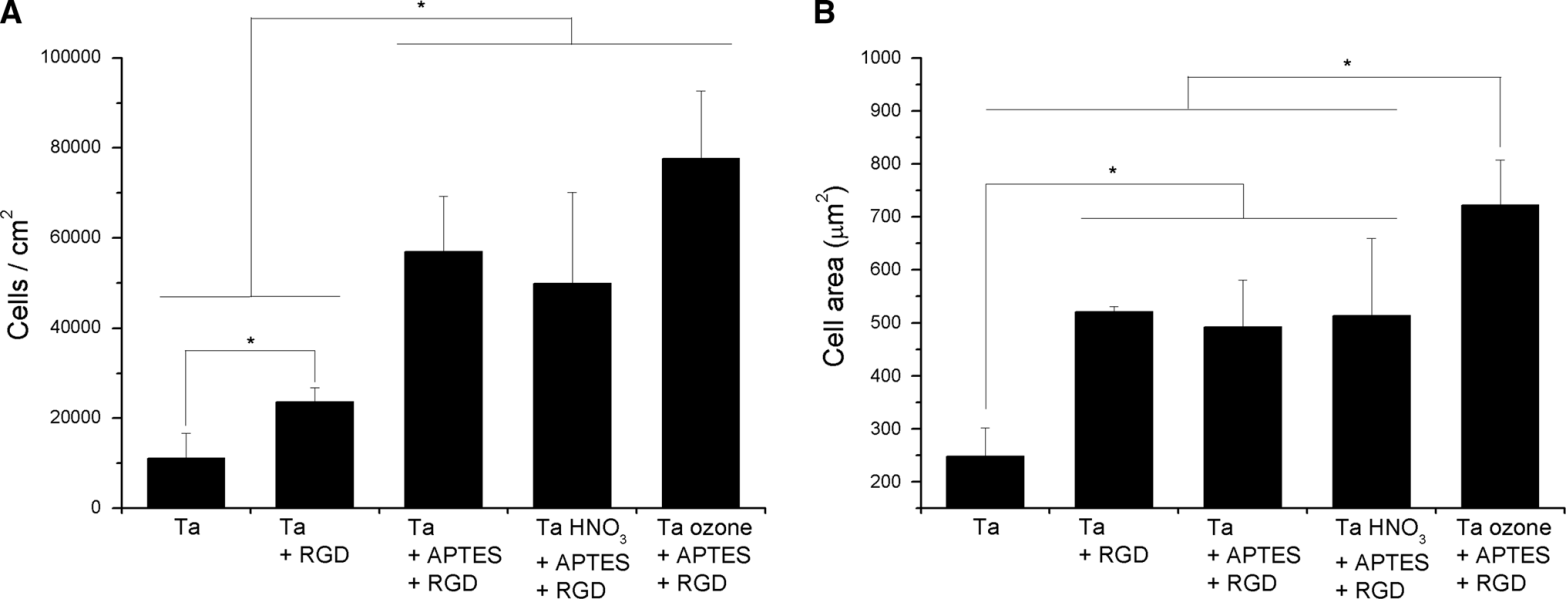
Adhesion of Saos-2 cells on Ta surfaces functionalized with the RGD peptide by silanization. The physisorption of RGD on Ta was also included as control. Cell adhesion was analyzed after 4 h of incubation. **a** Cell attachment (cells/cm^2^); and **b** cell spreading (cell area in µm^2^). Values are expressed as mean ± standard deviation. The symbol (*) denotes statistically significant differences between groups (cell numbers P < 0.1, cell area P < 0.05)

Silanization also proved useful to improve cell adhesion on Ta surfaces (Fig. 7). Interestingly, regardless of the activation method, silanization not only increased the number of cells attached compared to control Ta surfaces, but also compared to Ta + RGD surfaces (P < 0.1). Therefore, the covalent immobilization of the RGD motif would be a preferable approach than physical adsorption to improve the cell binding-capacity of Ta substrates. No significant differences were observed between the three methods of silanization, although a tendency towards increased cell numbers was observed for samples activated with ozone. This result supports the fact that activation with ozone yields the most efficient silanization of the surfaces and therefore presents a higher density of peptide on the surfaces. Cell spreading data corroborate that: RGD binding through physical adsorption or silanization statistically increased (P < 0.05) cell spreading in comparison with control Ta, however, silanization via ozone activation displayed the highest values of cell spreading (P < 0.05). Hence, conversely to physisorption, activation of surfaces with UV/ozone seems to be the best method for peptide attachment through silanization with APTES.

## Conclusion

In this work we have explored the functionalization of Ta samples with a cell adhesive peptide to improve Ta’s bioactivity. Two methods were studied: physical adsorption and covalent binding via silanization with APTES. Both methods were shown to be efficient in increasing the number and area of adherent cells. However, interesting differences were also observed. Whereas the physical adsorption of an RGD peptide was easily carried out on control and HNO_3_-treated Ta surfaces, binding of this peptide on highly hydrophilic, UV/ozone-treated Ta surfaces proved to be inefficient and had no effect in cell activity. On the contrary, UV/ozone-activated surfaces promoted the most efficient silanization and yielded the highest values of cell adhesion compared to the other strategies of silanization. Thus, both physical adsorption and silanization are feasible methods to anchor bioactive peptides on Ta surfaces, provided that the appropriate activation methods are used. Moreover, silanization methods displayed higher values of cell attachment than physisorption. Immobilization of peptides with other biofunctionalities (e.g. antibacterial, osteogenic properties, etc.) may be pursued to confer additional biological functions to Ta. These strategies could be easily applied to porous Ta implants and other Ta-based scaffolds to increase the osseointegrative properties of such materials.
